# Does finasteride treatment for benign prostatic hyperplasia influence sperm DNA integrity in dogs?

**DOI:** 10.1186/s12610-020-00108-2

**Published:** 2020-07-16

**Authors:** Daniel S. R. Angrimani, Luana C. Bicudo, Nuria Llamas Luceño, Bruno R. Rui, Matheus F. Silva, João D. A. Losano, Bart Leemans, Ann Van Soom, Camila I. Vannucchi

**Affiliations:** 1 Department of Animal Reproduction, School of Veterinary Medicine and Animal Science, University of São Paulo, Av. Prof. Orlando Marques de Paiva, 87, São Paulo, 05508-270 Brazil; 2 Department of Reproduction, Obstetrics and Herd Health, Faculty of Veterinary Medicine, Ghent University, 9820 Merelbeke, Belgium

**Keywords:** Prostate, Chien, Fragmentation de l’ADN, Spermatozoïdes, Finastéride, Prostate, Canine, DNA fragmentation, Sperm, Finasteride

## Abstract

**Background:**

Benign prostatic hyperplasia (BPH) is one of the most common reproductive disorders in both male dogs and men. Finasteride, a synthetic inhibitor of the enzyme 5α-reductase, is widely used as medical treatment. Although sperm can be affected by both BPH and finasteride treatment, the direct influence on DNA integrity remains unclear. Thus, the aim of this study was to verify the direct effect of BPH and/or finasteride treatment on DNA integrity of dog spermatozoa. A 2 × 2 factorial experiment was designed with 20 male dogs assigned to 4 experimental groups: BPH Group (*n* = 5), BPH-Finasteride Group (*n* = 5), Non-BPH Finasteride-Treated Group (n = 5) and Non-BPH Untreated Group (n = 5). Sperm evaluation was performed monthly for 60 days after the start of finasteride therapy or BPH diagnosis (D0, D30 and D60). Sperm DNA integrity was analyzed through fragmentation susceptibility (toluidine blue staining and Sperm Chromatic Structure Assay - SCSA), direct evaluation of DNA fragmentation (Sperm Chromatin Dispersion Assay - SCDA) and sperm protamination (chromomycin A3).

**Results:**

Sperm DNA integrity was not affected by finasteride treatment. However, BPH dogs had higher susceptibility to sperm DNA acid denaturation (SCSA) compared to dogs not presenting BPH, as well as lower percentage of sperm with DNA integrity (toluidine blue staining).

**Conclusion:**

In conclusion, benign prostatic hyperplasia causes post-testicular sperm DNA damage, albeit finasteride treatment itself does not directly influence sperm DNA integrity.

## Résumé

**Contexte:**

L’hyperplasie bénigne de la prostate (HBP) est l’un des troubles de la reproduction les plus courants chez le chien et chez l’homme. Le finastéride, un inhibiteur synthétique de l’enzyme 5α-réductase, est largement utilisé comme traitement médical. Bien que le sperme puisse être affecté à la fois par l’HBP et par le traitement avec le finastéride, l’influence directe sur l’intégrité de l’ADN reste peu claire.

Le but de cette étude était ainsi de vérifier l’effet direct de l’HBP et/ou du traitement par finastéride sur l’intégrité de l’ADN des spermatozoïdes de chien. Dans la présente étude, 20 chiens mâles ont été randomisés selon un plan factoriel en 2x2 à l’un des 4 groupes expérimentaux suivants : Groupe HBP (n=5), Groupe HBP-Finastéride (n=5), Groupe non-HBP traité par Finastéride (n=5), et Groupe non-HBP non traité (n=5). L’analyse Le sperme a été réalisée mensuellement pendant 60 jours (J0, J30 et J60) soit après le début du traitement par finastéride ou à partir du diagnostic de HBP. L’intégrité de l’ADN des spermatozoïdes a été analysée par l’évaluation de la susceptibilité à la fragmentation (coloration au bleu de toluidine ; détermination de la structure de la chromatine des spermatozoïdes - SCSA), par l’évaluation directe de la fragmentation de l’ADN des spermatozoïdes (détermination de la dispersion de la chromatine des spermatozoïdes - SCDA) et par l’évaluation de la protamination des spermatozoïdes (chromomycine A3).

**Résultats:**

L’intégrité de l’ADN des spermatozoïdes n’a pas été affectée par le traitement par finastéride. Cependant, les chiens avec HBP ont une susceptibilité plus élevée à la dénaturation acide de l’ADN des spermatozoïdes (SCSA) par comparaison aux chiens ne présentant pas d’HBP, ainsi qu’un pourcentage plus bas de spermatozoïdes avec intégrité de l’ADN (coloration au bleu de toluidine).

**Conclusions:**

L’hyperplasie bénigne de la prostate induit des altérations de l’ADN des spermatozoïdes, alors que le traitement par finastéride n’influence pas directement par lui-même l’intégrité de l’ADN des spermatozoïdes.

## Background

Benign prostatic hyperplasia (BPH) is a reproductive disorder of men and dogs [1, 2], with high prevalence in aged males [3, 4]. The etiology of BPH is related to a hormonal imbalance between testosterone and estrogen and an increased activity of 5α-reductase, leading to higher dihydrotestosterone (DHT) concentrations [5]. The overproduction of DHT induces prostatic cell proliferation, causing an abnormal enlargement of the prostate gland [6].

The hormonal imbalance and subsequent increase in prostate volume are associated with several reproductive clinical signs. Impaired spermatogenesis is often observed [7], which may affect sperm DNA integrity [8, 9]. Sperm DNA damage related to BPH was previously described in dogs [3] and men [4], albeit not yet completely elucidated. It is suggested that biochemical modifications of the prostatic fluid play an important role on sperm damage. Moreover, senescence and prostatic changes increase local oxidative stress, generating higher levels of reactive oxygen species and decreased antioxidant defense [10].

There are different forms to treat canine BPH. Besides the existence of potent antiandrogen drugs (e.g., osaterone acetate), their availability is restricted to certain countries, limiting a world-wide prescription [11–13]. In dogs, orchiectomy is the permanent way to remove the androgenic stimulation of BPH [14], whereas finasteride, a synthetic inhibitor of the 5α-reductase enzyme, is the most employed therapy for BPH in men [15]. Hence, finasteride can be used as an alternative for surgical treatment (orchiectomy) in breeding dogs with high genetic value, since previous studies have described little effect of finasteride on sperm quality (motility and morphology) and testosterone concentrations [16, 17]. Although Iguer-Ouada & Verstegen [18] have attested the fertility of dogs under 20 weeks of finasteride treatment, in men, finasteride causes oligospermia or azoospermia and sperm DNA fragmentation [19, 20], related to a deleterious effect on spermatogenesis [19–21]. In addition, Vidigal et al. [22] showed reduced spermatogenesis and seminiferous tubules atrophy in finasteride treated hamsters.

DNA fragmentation has a dramatic effect on fertilization rates, embryonic development and embryo implantation [23–25]. Sperm chromatin damage has been associated with spermatogenesis failure or post-testicular disorders, likely related to reactive oxygen species-induced damage [26]. Thus, a deep-screening of sperm DNA integrity can suggest more precisely the origin of the chromatin damage [24]. Therefore, the aim of this study was to characterize the effects of BPH and finasteride therapy associated with structural abnormalities of sperm DNA in dogs.

## Methods

### Animals and experimental study

This study was previously approved by the Bioethics Committee of the School of Veterinary Medicine and Animal Science - University of São Paulo (protocol number: 7122171213). All chemicals used in this study were obtained from Sigma-Aldrich (St. Louis, MO, USA) unless otherwise listed.

As described in detail previously, using the same study population [27], 20 male dogs of several breeds, aging from 5 to 13 years and weighting from 10 to 30 kg were selected for this study and assigned to four experimental groups:

1. BPH Group (*n* = 5): dogs with mean age of 10.8 years and body weight of 18.8 kg were presumptively diagnosed with BPH based on clinical signs (hematospermia, hematuria, pollakiuria, dysuria and tenesmus), marked prostatomegaly and prostatic biometry by B-mode ultrasound [28, 29].
2. BPH-Finasteride Group (*n* = 5): dogs with mean age of 9.2 years and body weight of 23.4 kg were presumptively diagnosed with BPH (as for the BPH Group) and subjected to BPH treatment with 5 mg finasteride per animal, orally, every 24 h for 2 months [30].
3. Non-BPH-Finasteride Group (*n* = 5): dogs with mean age of 7.4 years and body weight of 20.8 kg without BPH were subjected to finasteride treatment protocol (as for the BPH-Finasteride Group). The diagnosis of BPH was ruled out by the lack of characteristic clinical signs coupled with a normal prostate morphometry by B-mode ultrasonography [28, 29].
4. Non-BPH-Untreated Group (Control Group, *n* = 5): dogs with mean age of 7.2 years and body weight of 22.3 kg, not presenting BPH (as for the Non-BPH-Finasteride Group) nor subjected to finasteride protocol.

To assure the appropriate sample size, an analysis was conducted with the SAS Power and Sample Size 12 (SAS Institute Inc., Cary, NC, EUA). A retrospective analysis of the data indicated there was a power of 0.99, which is considered an acceptable statistical power (at least 0.8). Hence, a minimum of 5 dogs per group were sufficient to demonstrate significant differences in the data.

### Seminal collection

Semen samples were collected monthly for 60 days, i.e., Day 0, Day 30 and Day 60, considering day 0 as the first day of finasteride treatment or BPH diagnosis. Semen was collected by penile digital manipulation directly into a calibrated plastic tube connected to a funnel. The sperm-rich fraction was collected by means of visual inspection of the ejaculate, as well as part of the 3rd fraction containing prostatic fluid. While collecting the sperm-rich fraction, we also included part of the prostatic fraction, as to achieve an equal proportion 1:1 of semen:prostatic fluid (v:v).

Subsequently, conventional sperm motility (%) was assessed under light microscopy (Nikon, Eclipse E200, Japan) at 400× magnification using 5 μL of semen placed on a pre-warmed glass slide with coverslip. Only dogs that showed total motility higher than 60% were included in this study. Then, the ejaculate was processed for sperm DNA integrity analysis.

### Evaluation of sperm susceptibility to DNA fragmentation

#### Toluidine blue (TB) staining

Semen smears were prepared and subjected to toluidine blue staining according to a protocol previously described by Rui et al. [31]. Smears were prepared with 10 μL of the sperm sample on a glass slide and subsequently fixed in 96% ethanol-acetone for 30 min at 4 °C. After drying, smears were hydrolyzed in 0.1 N HCl for 5 min at 4 °C and washed three times in distilled water for 2 min. Subsequently, smears were exposed to toluidine blue stain (0.05%) for 20 min and washed 2 times in distilled water for 2 min. Smears were evaluated under light microscopy (Leitz, Dialux 20, Germany) at 1000x magnification in a single-blind way, i.e., researchers were kept ignorant of either the group they were assessing. Sperm cells with damaged DNA were stained in blue, whereas intact DNA sperm remained unstained. For each sample, a minimum of 200 sperm cells were assessed and results were expressed in percentage (%) of DNA damaged spermatozoa.

#### Sperm chromatic structure assay (SCSA)

The assay was performed following the methodology adapted by Lucio et al. [32] for dogs, based on a protocol that allows the estimation of chromatin susceptibility to acid denaturation [33]. Chromatin instability after acid exposure was quantified by flow cytometer after acridine orange (AO) labeling. Based on the DNA integrity status, a metachromatic fluorescence shift is induced from green (double-strand DNA) to red (denatured single-strand DNA). We used the Guava EasyCyte™ Mini System (Guava® Technologies, Hayward, CA, USA), with a 488 nm argon laser and the following photodetector filters: PM1 (583 nm; yellow fluorescence), PM2 (680 nm; red fluorescence) and PM3 (525 nm, green fluorescence). A total of 20,000 sperm cells were considered from each sperm sample and data were analyzed using Flow Jo v8.7 Software (Flow Cytometry Analysis Software – Tree Star Inc., Ashland, Oregon, USA).

In brief, sperm samples (15 μL) were diluted in 100 μL TNE buffer (0.01 M Tris–HCl, 0.15 M NaCl, 1 mM EDTA, pH 7.4) and subsequently mixed with 400 μL of an acidified detergent solution (0.08 M HCl, 0.1% Triton X-100, 0.15 M NaCl, pH 1.2). After 30 s, sperm cells were stained by adding 600 μL of AO staining solution (0.037 M citric acid, 0.126 M Na_2_HPO_4_, 0.0011 M disodium EDTA, 0.15 M NaCl, pH 6.0). After 5 min, samples were examined by flow cytometry as described above. DNA fragmentation rates were calculated based on the percentage of spermatozoa outside the main population in an αT histogram (ratio between red fluorescence and total fluorescence) as evaluated using the Flow Jo system (Version Mac) [32, 34].

### Identification of DNA protamination by chromomycin A3 (CMA3)

In order to identify defects on sperm protamination process, the chromomycin A3 technique was performed based on protocols previously described by Rahman et al. [35] and Simões et al. [36]. A positive control sample of deprotaminated dog sperm was prepared by exposing spermatozoa to a solution of 0.001% Triton X-100 and 5 mM DTT in 200 μL phosphate buffered saline (PBS) for 15 min, followed by incubation in a solution of 1 M NaCl and 5 mM DTT in H_2_O for 2 h. Subsequently, the positive control and all other tested sperm samples were washed in PBS and fixed in Carnoy’s solution (3:1 methanol:acetic acid; Merck, Darmstadt, Germany) at 4 °C for 10 min. Then, smears were prepared and subsequently treated with 12.5 μL CMA3 solution [0.25 mg/ml in 1000 μL of McIlvaine buffer (7 ml of 0.1 M citric acid + 32.9 ml of Na_2_ HPO_4_ .7H_2_O, 2 M, pH 7.0, containing 10 mM MgCl_2_)] for 20 min. The slides were additionally stained for 2 to 5 min with Hoechst 33342 (5 mg/ml) in order to determine the total number of sperm cells. Then, slides were washed in PBS. Microscopic images were captured using fluorescence microscopy (Leica DMR, 400x magnification) with appropriate filters (460–470 nM) in a single-blind manner. A minimum of 200 cells were counted and results were expressed in percentage of sperm showing protamine deficiency (%).

### Direct verification of DNA fragmentation by the modified sperm chromatin dispersion assay (SCDA)

**Fig. 1:**
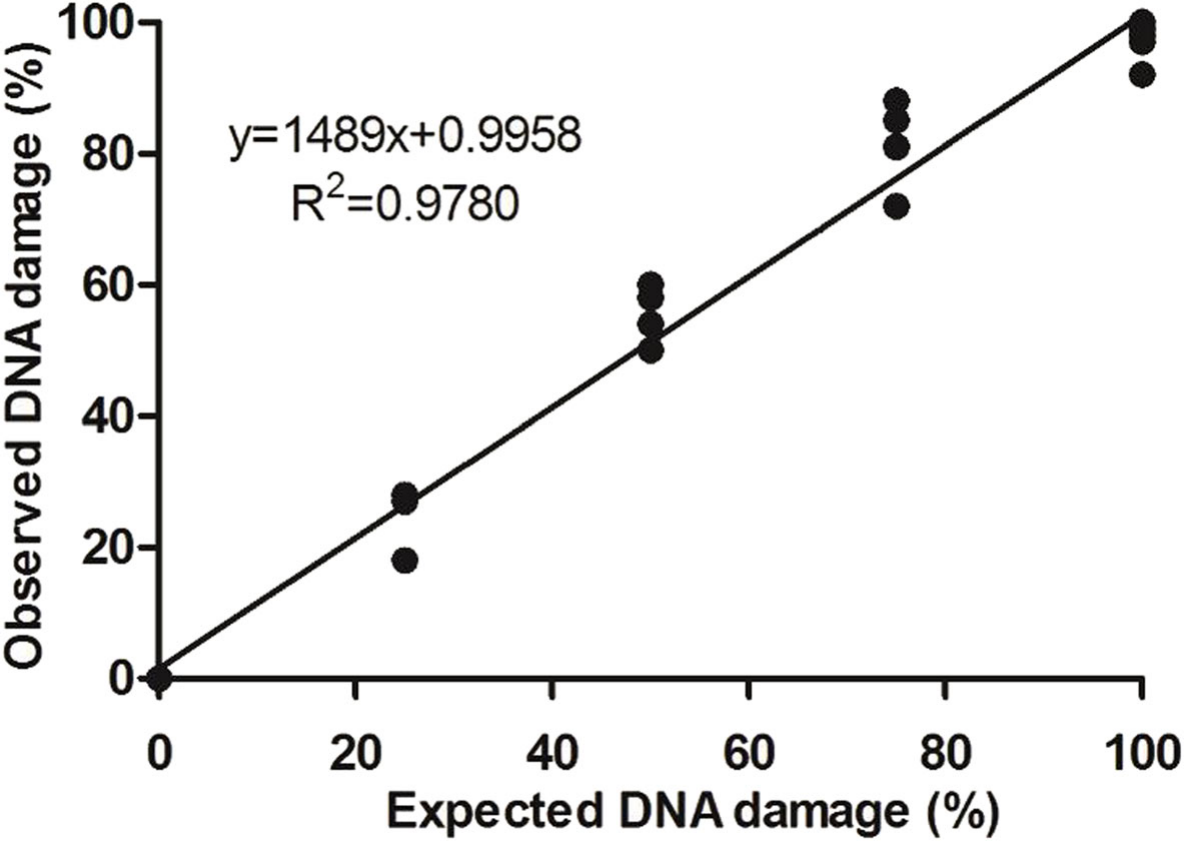
Linear regression analysis for the sperm chromatin dispersion assay (SCDA) validation for dog sperm

The assay was performed according to the procedure of Shanmugam et al. [37] and Fernández et al. [38]. Initially, the protocol was validated with the use of the sperm-rich fraction collected by digital manipulation from six sexually mature dogs of several breeds. All dogs had proven semen quality, confirmed by previous breeding soundness examination. Only sperm-rich fractions with a minimum of 60% total sperm motility were used. After sperm collection, one aliquot of each sample was kept at 5 °C and the remaining sample was exposed to ultraviolet light (Fluxo Veco VLFS-12 M, Campinas, São Paulo) for 4 h at 25 °C in order to artificially induce DNA fragmentation. Both aliquots were then mixed to obtain different known proportions of fragmented DNA sperm (0, 25, 50, 75 and 100%). Sperm smears were subsequently prepared on a glass slide with 10 μL of each mixture of damaged/intact DNA sperm. Evaluation of DNA fragmentation was performed using the modified chromatin dispersion assay (SCDA). A minimum of 200 sperm cells were counted and results were expressed as percentage (%) of DNA fragmented spermatozoa in a single-blind way, i.e., researchers were kept ignorant of either the group they were assessing. A high linear regression coefficient (R^2^ = 0.97, *p* = 0.001) between the observed and the expected percentages of spermatozoa exhibiting DNA damage attested the validation of the SCD assay for dogs (Fig. 1).

For each sperm sample of the present experiment, equal volumes (1:1) of diluted semen (1 × 10^6^ sperm/mL) and 1% low-melting agarose were mixed at 37 °C. An aliquot of 10 μL of this mixture was pipetted on a glass slide coated with 0.65% normal melting point agarose and subsequently covered with a cover slip and kept for 10 min at 4 °C in order to solidify. Immediately after careful removal of the cover slip, the slides were immersed horizontally for 3 min at 22 °C in the dark in a tray containing acid denaturation solution (0.08 N HCl). This condition generates restricted single-stranded DNA (ssDNA) motifs from DNA breaks. Subsequently, the denaturation was stopped and proteins were removed by transferring the slides for 2 h at 4 °C to a tray with neutralizing and lysis solution (10 mM Tris, 4 mM DTT, 2% Triton X-100, 100 mM Na_2_ - EDTA, 2.5 M NaCl, pH 11). The slides were washed in Tris-borate-EDTA buffer (0.09 M Tris-borate and 0.002 M EDTA, pH 7.5) for 2 min, dehydrated in sequential 70, 90 and 100% ethanol (2 min each) and, then, air dried. Prepared slides were horizontally covered with a mix of Wright’s stain and buffer solution (380 mg Na_2_ PO_4_, 547 mg KH_2_ PO_4_ in 100 mL distilled water) for 10 min with continuous air flow. The stain was poured off and slides were briefly washed in tap water and dried.

The stained slides were evaluated under a light microscope and 200 sperm were evaluated per slide for halo size and dispersion pattern at 1000× magnification in a single-blind manner. The nuclei with large to medium halo size were considered sperms with non-fragmented DNA, while nuclei with small halo size or without halo were considered as sperm cells with fragmented DNA.

### Statistical analysis

All data were evaluated using SAS System for Windows (SAS Institute Inc., Cary, NC, USA). Effects of BPH, finasteride, moment of evaluation (days 0, 30 and 60) and interactions between these factors, were estimated by repeated measures analysis of variance (Mixed Procedure of SAS). If no triple interactions (BPH X finasteride X Timing) existed, the following interactions were considered: Timing *x* finasteride, Timing *x* BPH and finasteride *x* BPH. If no dual significant interactions were observed, then effects of groups were analyzed by merging all time points and conversely, time points were compared by combining all groups; otherwise, comparisons were performed taking both effects into account. Differences between BPH and finasteride treatment were analyzed using parametric and non-parametric tests, according to the residual normality (Gaussian distribution) and variance homogeneity. Data were transformed if one of these assumptions was not respected. When transformations were not successful, non-parametric tests were used. Moreover, differences between BPH and finasteride treatment were analyzed using Student t-test (parametric variables) and Wilcoxon test (nonparametric variables). Results were described as untransformed means ± SE. The significance level was *P* < 0.05.

## Results

No significant triple or dual interaction between time points, BPH diagnosis and finasteride treatment were observed. Hence, the effect of groups was analyzed merging all time points, with special reference to the comparison between BPH and Non-BPH (irrespective of finasteride treatment) and Finasteride and Untreated dogs (regardless of BPH diagnosis).

**Fig. 2:**
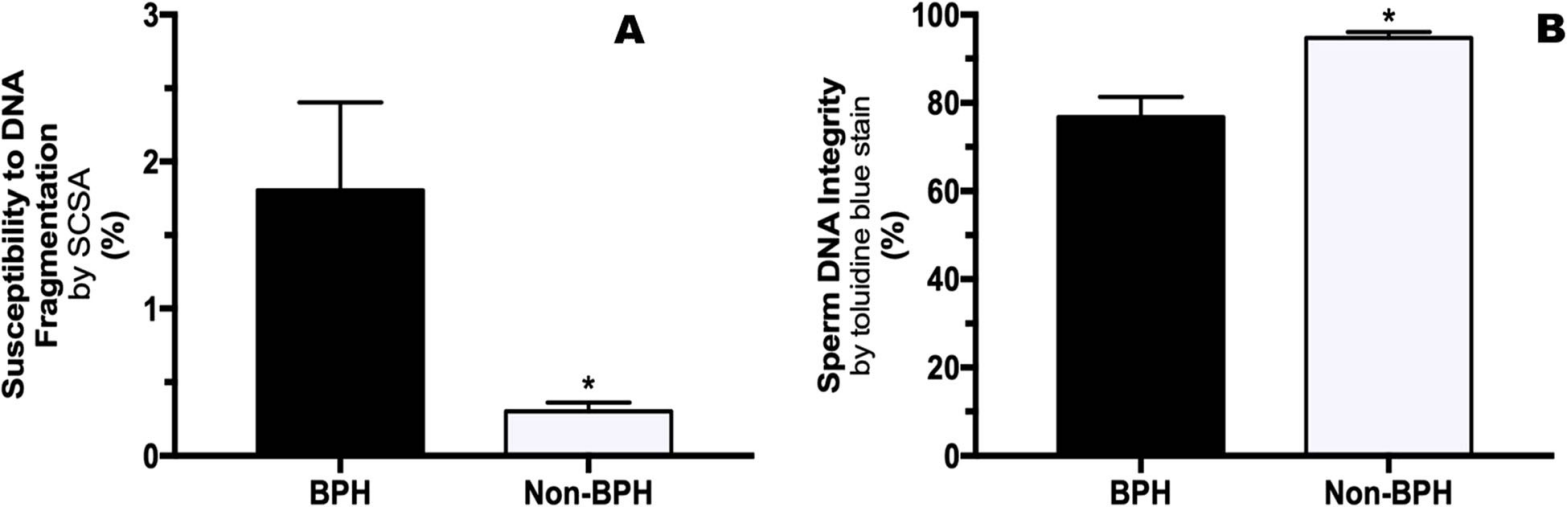
(**a**) Susceptibility to DNA fragmentation analysed by the Sperm Chromatic Structure Assay (SCSA) and (**b**) Toluidine Blue Stain in the BPH and Non-BPH Groups. *indicate significant difference between groups (P < 0.05)

Sperm DNA integrity was not different between Finasteride and Untreated dogs (Table 1) and time points (Day 0 vs. Day 30 vs. Day 60). However, BPH dogs had higher susceptibility to DNA fragmentation (1.86 ± 0.69%) compared to Non-BPH dogs (0.3 ± 0.06%; Fig. 2a). In addition, BPH dogs had higher percentage of DNA fragmentation sperm (23.2 ± 4.6%), compared to the Non-BPH Group (5.3 ± 1.3%, Fig. 2b). No significant differences between BPH and Non-BPH dogs were observed for sperm chromatin dispersion assay (SCDA) and CMA3 (Table 2).

**Table 1:** Mean ± SE of DNA damaged sperm (%) of the Finasteride and Untreated Groups

	Finasteride Treated	Untreated	P
SCD	12 ± 2.9	14.4 ± 2.4	0.42
SCSA	1.8 ± 0.7	0.4 ± 0.07	0.07
TB	17.5 ± 3.9	11.2 ± 3.6	0.11
CMA3	0.5 ± 1.2	1.8 ± 1.6	0.16

*SCD* sperm chromatin dispersion, *SCSA* sperm chromatic structure assay, *TB* toluidine blue stain, *CMA3* chromomycinA3

**Table 2:** Mean ± SE of DNA damaged sperm (%) in the BPH and Non-BPH Groups

	BPH	Non-BPH	P
SCDA	15.8 ± 2.9	10.5 ± 2.1	0.42
CMA3	8.3 ± 0.8	9.4 ± 1.1	0.44

*SCD* sperm chromatin dispersion, *CMA3* chromomycinA3

## Discussion

In this study, we have assessed the effects of both Benign Prostatic Hyperplasia (BPH) and finasteride therapy for two consecutive months on sperm DNA integrity in dogs. Despite the relevance of finasteride treatment as an alternative for orchiectomy, only few studies have reported simultaneously the effects of BPH and finasteride on DNA integrity of dog sperm, leading to insecurity of employing such treatment in BPH breeding dogs.

In men, the use of finasteride causes adverse sperm DNA changes as an important side effect [20]. Conversely, our study demonstrated that the 2 months course of finasteride treatment was not able to cause DNA damage in dog sperm, by means of an overall tracking analysis with SCDA, CMA3, TB and SCSA. We suggest that finasteride detrimental effects in men are related to a dose dependent action, since finasteride posology in dogs is considered to be lower than the one preconized in men, and the time-course of treatment shorter [39]. Thus, we can affirm that the current finasteride protocol (2 months course of 5 mg/dog) can be safely used without additional adverse effects on sperm DNA integrity, while assuaging clinical signs and reducing prostate size [27]. Additionally, finasteride is recognized to be innocuous for sperm motility and morphology after 4 months of treatment [17]. These data suggest that dogs under finasteride therapy can be safely employed in reproductive programs. However, it is important to point out that a proven fertility screen after finasteride treatment can only be attested if other sperm quality parameters are examined simultaneously [40]. It is of utmost importance to evaluate sperm membrane and acrosome integrity, in addition to chromatin status [41–43].

Albeit finasteride treatment was not able to adversely affect sperm DNA integrity, we showed that BPH dogs have high susceptibility to sperm DNA fragmentation, as observed by SCSA and TB assays. The current finding is of practical importance, since sperm DNA denaturation may be responsible for infertility, abortion, and foetal malformations [30]. Flores et al. [3] and Krakowski et al. [8] have also observed high chromatin instability and susceptibility to sperm DNA fragmentation in BPH dogs. Although several conditions may lead to sperm DNA damage [44], changes in the composition of the prostatic fluid are considered one of the main reasons in BPH dogs [8]. In men, Zabaiou et al. [45] showed that BPH is responsible for oxidative changes of the prostate tissue, accompanied by reduction in prostatic antioxidant concentrations. Accordingly, BPH may also modify the oxidative status of the canine prostatic fluid. In fact, Krakowski et al. [8] showed that the prostatic fluid of BPH dogs has important biochemical alterations, such as high pH, increased cholesterol and decreased zinc and copper concentrations. Zinc deficiency has been associated with DNA fragmentation and oxidative stress, since zinc is a component of the superoxide dismutase, an important antioxidant regulating enzyme [8, 46, 47]. The spermatozoa itself can be a source of reactive oxygen species and, therefore, an autologous adverse effect is triggered. In fact, Vieira et al. [48] reported that oxidative stress is related to sperm mitochondrial malfunction in dogs. Additionally, reduction in sperm mitochondrial activity was observed in BPH dogs [3]. Taken these data together, we assume that BPH is responsible for sperm mitochondrial damage, which increases local oxidative stress, ultimately, inducing sperm DNA fragmentation.

Regarding the source of the sperm DNA damage, it is possible to exclude a testicular origin (during spermatogenesis) of the higher susceptibility to sperm DNA fragmentation due to deprotamination [44]. The replacement of sperm DNA histones by protamines results in a more condensed DNA compared to somatic cells [49, 50], consequently higher DNA resistance to damage by external chemical agents and radiation [51]. Since we could not verify differences in CMA3 labelling (i.e., identification of protamine deficient cells) among experimental groups, we suggest that increased susceptibility to sperm DNA fragmentation in BPH dogs is not caused by testicular failure of DNA packaging during spermatogenesis and may occur in a post-testicular environment, for example, after the exposure to the seminal plasma [26]. However, further studies are needed in order to evaluate the long-term action of finasteride farther than 65 days, as to compromise a full canine spermatogenic cycle.

In the present study, SCD assay was successfully validated for dogs and further employed to directly evaluate sperm DNA breaks [52], using only light microscopy and easily available chemical reagents, making such analysis accessible to most laboratories. SCD assay allows identifying physical DNA breaks followed by a problem during spermatogenesis or testicular degeneration [26]. However, no difference in SCDA among groups existed in the present experiment, reaffirming the lack of testicular influence on sperm DNA fragmentation in BPH dogs. Thus, sperm derived from BPH dogs does not show protamination failure or direct DNA damage, albeit a higher susceptibility to DNA fragmentation, which may indicate a role of local alterations of the prostatic fluid. Hence, these data should be taken into account while inducing additional sperm stress as, for example, during sperm cryopreservation and cooling [48].

## Conclusion

In conclusion, sperm of BPH dogs are highly susceptible to DNA fragmentation, which is much likely derived from post-testicular changes of the prostatic fluid. In addition, finasteride treatment is not able to provoke any additional sperm DNA damage. Thus, finasteride can be safely applied in a 2 months course for BPH treatment in dogs, in respect to sperm DNA integrity. To the best of our knowledge, this is the first report to describe the effects of finasteride on sperm DNA integrity in dogs. However, future studies should address the effect of finasteride on an overall panel of sperm quality, including the analysis of sperm plasma membrane and acrosome integrity in BPH dogs, as well as sperm analysis farther than 65 days, trying to reach accurately the entire canine sperm cycle.

## Data Availability

All data generated and analysed during thus study are included in this published article.

## Abbreviations

AO: Acridine orange
BPH: Benign prostatic hyperplasia
CMA3: Chromomycin A3
DHT: Dihydrotestosterone
SCSA: Sperm chromatic structure assay
SCDA: Sperm chromatin dispersion assay
TB: Toluidine Blue
